# Primary care providers’ perspectives of experiences with transgender and gender diverse adults: A scoping review

**DOI:** 10.1080/13814788.2026.2657083

**Published:** 2026-04-20

**Authors:** Kate Lauren Keaney, Marie Claire Van Hout, Geoff McCombe, Nandakumar Ravichandran, John Broughan, Walter Cullen, Des Crowley

**Affiliations:** a School of Medicine, University College Dublin, Dublin, Ireland; b Office of the Vice President of Research, Innovation and Impact, South East Technological University, Waterford, Ireland; c Health Service Executive, Addiction Services, Dublin, Ireland; d Addiction Management in Primary Care Division, Irish College of General Practitioners, Dublin, Ireland

**Keywords:** Primary care provider, transgender and gender diverse, healthcare barriers

## Abstract

**Background:**

As a minority group, transgender and gender diverse (TGD) individuals may experience healthcare stressors due to stigma, transphobia and healthcare staff who lack the knowledge about their specific needs, impacting primary care entry and attendance. Extant literature is concentrated on TGD individuals’ perspectives of primary care with the main message being that it is inadequate.

**Aim:**

To explore the primary care provider (PCP) perspective of experiences with adult TGD patients.

**Methods:**

A scoping review was conducted using Arksey and O’Malley’s five-step framework to map and de-scribe the literature relating to PCP perspectives’ of experiences with TGD adults. Four databases were searched: PubMed, Embase, PsycINFO and CINAHL Plus. The process was guided by the Preferred Reporting Items for Systematic Reviews and Meta-Analyses extension for Scoping Reviews (PRISMA-ScR). The final dataset (*n* = 22) were charted and analysed thematically.

**Results:**

Following application of exclusion criteria and removal of duplicates, twenty-two studies across five countries were included in the review. Studies include qualitative studies, surveys, mixed methods studies, a retrospective review and a pilot study with a pre- and postintervention analysis. Key themes identified related to the attitudes of PCPs towards TGD individuals, education of PCPs on TGD health issues and the barriers/facilitators to caring for TGD patients from a PCP perspective.

**Conclusion:**

The review highlights the need for improved access to TGD-specific education. Future research should explore the best way to provide this to PCPs, inform implementation into relevant policies and include a broader range of countries to strengthen global applicability.

## KEY MESSAGES

- Lack of TGD-specific healthcare knowledge, swtemming from educational deficits, is the primary barrier to care.
- Education programmes and digital interventions (e.g. eConsults), improve PCP comfort and quality of care.
- Future research should identify optimal methods of delivering training to PCPs and inform implementation into relevant policies.

## Introduction

Transgender (trans) is an umbrella term referring to people ‘whose gender identity and/or gender expression differs from the sex assigned to them at birth’ [1]. Trans people may have a binary gender which differs to their sex assigned at birth (male or female), or they may have a gender identity which falls outside of this (non-binary). Cisgender people have a gender identity which aligns with their sex assigned at birth [1].

Transgender and gender diverse (TGD) individuals make up a substantial and increasing proportion of the population, with the exact percentages depending on different inclusion criteria (e.g. age or definition of trans) ranging from around 1–8% [2,3]. TGD individuals have unique healthcare needs with higher rates of mental health problems, substance abuse, cardiovascular disease, sexually transmitted infections, human immunodeficiency virus (HIV) and suicide attempts in comparison to the cisgender population [2], along with TGD-specific needs including gender-affirming care (GAC) [4,5]. With primary care being the first healthcare interaction, it plays a critical role in providing safe, supportive, sensitive and non-judgmental care to the TGD population [2,6].

As a minority group, TGD individuals may experience increased healthcare stressors due to stigma, transphobia, discrimination and healthcare staff who lack the knowledge about them and about how to care for them, etc [2,4,5,7,8]. These factors impact attendance to primary care, causing a lower uptake of services such as cancer screenings [7,9].

TGD adults in high-income countries encounter inadequately trained primary care providers (PCPs) and discrimination when seeking primary care [2,4,5,7,8]. While systemic issues exist, positive experiences with certain providers improved healthcare engagement and reduced psychological distress [7]. PCPs could provide better support by advocating on behalf of TGD patients, engaging in greater communication with the local TGD community and staying up to date with guidelines and research [4,8]. Evidence shows that there was a desire for more primary care-led services to initiate, follow-up and support all stages of GAC, as well as having more TGD representatives in clinic [8].

To our knowledge, a literature review of TGD primary care from the perspective of the PCP is lacking. Extant literature is concentrated on TGD individuals’ perspectives of primary care, with the main message being that it is inadequate [8]. Exploring the consultation from the PCP perspective will add some of the missing evidence to implement the necessary changes and support enhanced PCP sensitisation and competencies in caring for TGD patients. A scoping review was deemed the most suitable methodology as the purpose of this paper was to map and describe existing literature regarding the PCP perspectives of experiences with TGD adults, identify key concepts and gaps within the topic and inform future policy, practice and research.

## Methods

The scoping review was conducted from mid 2024 to mid 2025 and used the five-stage framework developed by Arksey and O’Malley [10]. A study protocol was not produced for this review.

### Step 1: Identifying the research question

The review was underpinned by two central research questions: 1) What are the PCP perspectives of experiences with TGD adults internationally? 2) What are the main barriers and facilitators to care, from these PCP perspectives?

### Step 2: Identifying relevant studies

A preliminary search of key databases was performed on 21 May 2024. Multiple search terms were used to generate a reading list. Keywords were identified, and medical subject heading (MeSH) terms were generated from this list. A more comprehensive search was carried out on 24 June 2024. The final search was carried out on 29 July 2025, the search included in this scoping review (Table 1). The electronic databases searched were PubMed/MEDLINE, Embase, CINAHL Plus and APA PsycINFO.

**Table 1.** Search strategy.

PubMed/MEDLINE	Embase
(‘Transgender’ OR ‘gender non-conforming’ OR ‘gender nonbinary’ OR ‘transgender persons’[MeSH]) AND (‘general practitioner’ OR ‘family physician’ OR ‘primary care provider’ OR ‘family doctor’ OR ‘family physician’ OR ‘primary care doctor’ OR ‘primary care physician’ OR ‘Physicians, Primary Care’[Mesh] OR ‘General Practitioners’[Mesh])	(‘Transgender’ OR ‘gender non-conforming’ OR ‘gender nonbinary’ OR ‘transgender and gender nonbinary’/exp) AND (‘general practitioner’ OR ‘family physician’ OR ‘primary care provider’ OR ‘family doctor’ OR ‘family physician’ OR ‘primary care doctor’ OR ‘primary care physician’ OR ‘general practitioner’/exp)
CINAHL Plus	APA PsycINFO
(‘Transgender’ OR ‘gender non-conforming’ OR ‘gender nonbinary’) AND (‘general practitioner’ OR ‘family physician’ OR ‘primary care provider’ OR ‘family doctor’ OR ‘family physician’ OR ‘primary care doctor’ OR ‘primary care physician’)	(‘Transgender’ OR ‘gender non-conforming’ OR ‘gender nonbinary’) AND (‘general practitioner’ OR ‘family physician’ OR ‘primary care provider’ OR ‘family doctor’ OR ‘family physician’ OR ‘primary care doctor’ OR ‘primary care physician’)

*Notes:* Search filters: 2014–2025, ‘Apply equivalent subjects’ expander on CINAHL Plus and APA PsycINFO.

### Step 3: Study selection

Initial searching and screening were conducted by one reviewer (KLK) with input from the research team (DC, MCVH, GMC). Duplicates were removed, and the literature was subsequently screened by title and/or abstract (KLK). Studies deemed relevant were selected for a full-text review performed independently by two reviewers (KLK, DC). Inclusion criteria were broad to cover a wide range of literature, consistent with the Arksey and O’Malley framework [10], as seen in Table 2.

**Table 2.** Selection criteria.

Inclusion criteria	Exclusion criteria
Full text published in English	No full text available in English
Published from 2014 to 2025	Published before 2014
Adult patient population (over 18 years old)	Paediatric patient population (under 18 years old)
PCPs’ perspectives of interactions with TGD patients	No PCP perspectives of interactions with TGD patients

*Note:* PCP: primary care provider; TGD: transgender and gender diverse.

### Step 4: Charting the data

Once the relevant literature was identified, data were organised into a table to facilitate analysis and comparison by KLK (see Table 3). Data were charted according to the framework of Arksey and O’Malley [10].

**Table 3.** Studies included in the review.

Citation details	Location	Study population	Study aim	Study design	Outcome measures	Key findings	Implications for policy, practice and research
Akre et al. [12]	USA	*N* = 1245 primary care practices (primary care physicians and practice managers)	To assess the engagement of primary care practices in LGBTQ+ affirming care activities and identify practice characteristics associated with increased engagement.	Cross-sectional study using a survey	SOGI-affirming activities	High rates of SOGI data collection but limited use of these data to improve performance or train staff. 34.42% provided LGBTQ+ training for clinicians and 39.20% for staff. Practices with FQHC-status, with a Medicaid payer mix at least 50% and areas with higher state-level LGBTQ+ Equality Score all had higher probability of engaging in all SOGI-affirming activities. Southern and rural areas had the lowest probability of having higher performing practices.	‘If the Liaison Committee for Medical Education and the Accreditation Council for Continuing Medical Education included LGBTQ+ health training as a requirement for accreditation, it could reduce misinformation contributing to stigmatization and discrimination of LGBTQ+ patients in health care and increase the confidence and proficiency of clinicians so that LGBTQ+ affirming care could be more broadly considered a standard of all care rather than just specialty care.’ [12]
Beagan et al. [13]	Canada	*N* = 24 family physicians	To analyse the experiences and understandings of GPs in two Canadian cities about their work with women patients who identify as LGBTQ, focusing on rationales for how they worked with LGBTQ patients and examining when/how SOGI were deemed to matter.	Qualitative study comprised of interviews.	The extent to which SOGI matters/makes a difference for physicians caring for LGBTQ patients.	Three major themes were identified: (1) SOGI makes little/no difference; treating every patient as an individual and avoiding labels optimises care, (2) SOGI matters primarily for the provision of holistic care and to address the effects of discrimination, (3) SOGI both matters and does not matter, the implications of social group membership must be balanced with the recognition of individual differences.	‘Sexual/gender identity matters little if at all means physicians may be ignoring important aspects of social group memberships that affect health and health care. Helping students to understand the importance of generalizations and the difference from stereotyping, may be an important addition to curricular efforts to improve education concerning LGBTQ health care.’ [13]
Bos and Bos [14]	Netherlands	*N* = 10 GPs and *N* = 15 TGD individuals	What factors in interactions between TGD individuals and GPs, in the Netherlands, hamper or facilitate health in- formation exchange?	Exploratory qualitative study using interviews and focus groups.	Addressing patients, knowledge and education, system and office environment, peers and society, moderation and mediation on health care utilisation	TGD individuals and GPs alike face similar and unique barriers when communicating with each other. The main findings of this study show that GPs and GP assistants incorrectly addressing TGD individuals, patient registration and documentation systems, unwelcoming GP office environment, lack of GP education on TGD-specific health information and TGD patient expectations were the main factors influencing GP-TGD interactions.	‘To facilitate appropriate health communication, GPs are advised to ask patients about their pronouns, adjust intake forms and put up TNB-specific posters or a Pride flag in their offices. TNB people are advised to make an introductory appointment to discuss their gender identity. Lastly, TNB topics should be included in medical curricula and online GP resources.’ [14]
Carroll et al. [15]	USA	*N* = 95 PCPs	To assess the level of familiarity and knowledge PCPs have with breast cancer screening recommendations for TGD patients and if there is variability of these levels based on the PCPs prior education and experience.	Electronic survey using REDCap	Demographics and training, familiarity with TGD breast cancer screening recommendations, confidence levels with screening recommendations, screening recommendation knowledge test, future TGD-specific training	The study revealed a lack of PCP familiarity and knowledge of breast cancer screening in TGD patients. Only 35% were aware that recommendations for breast cancer screening in TGD patients existed. Awareness increased with 6 or more hours of TGD-specific education and previously caring for 6 or more TGD patients, suggesting that increased TGD-specific education and clinical experience is important for developing better awareness. Younger practitioners place a higher importance on TGD-specific educational training. Respondents who identified as members of the LGBTQI+ community exhibited greater awareness compared to their non-LGBTQI+ counterparts.	‘Up-to-date breast cancer screening recommendations for TGD patients should be readily available across multiple platforms, target key audiences and integrated into transgender health educational curriculums to maximize awareness of these important recommendations.’ [15]
Christopherson et al. [16]	Canada	*N* = 188 family physicians, family medicine residents and NPs	To investigate if family physicians, family medicine residents and NPs in Saskatchewan lack comfort and knowledge about providing medical care to TGD patients, specifically TGD-related care, also, to see if a subset of these providers were interested in further training for optimal care of these patients.	Cross-sectional survey collected online using REDCap	Survey included: descriptive statistics, previous TGD-related healthcare training, have seen a TGD patient, comfort in providing care to TGD patients, reservations about providing care to TGD patients, awareness of LGBTQ+ safe space signage, answering questions regarding TGD cases, interest in further training on TGD care…	While 96% of PCPs were comfortable providing non-transition-related medical care, only 30% felt comfortable providing transition-related medical care to patients who are TGD. Less than a third of participants indicated having previous training in TGD-related care, which may be a contributing factor to PCPs lacking the necessary knowledge to comfortably provide transition-related care. Interest in future training is high and future educational interventions could help address this gap. The study is limited by its single geographical location, low response rate (13.5%) and potential self-report bias.	‘A future study could compare the comfort and knowledge of practitioners before and after completion of this training in our rural Canadian context. The patient/community perspective would also be important, pre- and post-training, to assess if the initiatives are attaining the goal of culturally safe and appropriate care.’ [16]
De Brosse et al. [17]	USA	*N* = 90 PCPs and *N* = 13 TGD people	To understand expectations TGD people have and compare those expectations with PCP readiness to provide such care.	Mixed-methods study (focus groups for TGD participants and survey for PCPs)	Positive and negative interactions with PCPs from TGD perspective, what TGD patients want PCPs to know to better meet their healthcare needs, TGD education received by PCPs, transphobia scale, PCP comfort addressing healthcare needs of TGD patients, PCP prescribing of GAHT, comparison of both groups.	Medical gatekeeping and traumatic medical encounters create barriers to primary care for TGD people which could be prevented with better PCP education around TGD community expectations. PCP survey response rate was 35%. Forty six percent had received ≤1 h of TGD education. While 98% of PCPs had cared for a TGD patient, 38% felt comfortable addressing the healthcare needs of TGD patients and a few held highly overtly transphobic views. Efforts to expand access to GAC should include PCP education on clinical knowledge gaps.	‘Efforts to expand access to gender-affirming primary care should include PCP education on clinical knowledge gaps. Humanizing TGD patient lived experiences and expectations may also help with the disconnect between TGD patients’ needs for competent care, and what PCPs are willing (or able) to provide.’ [17]
Domalaon et al. . [18]	USA	*n* = 180 and *n* = 386 military-affiliated family physicians	To assess changes in military-affiliated clinicians’ perspectives towards GAC over time	Serial cross-sectional survey	Participants’ perception of, comfort with, and education on GAC, comparing cohorts from 2016 and 2023.	Compared to 2016, clinicians in 2023 were significantly more likely to report receiving relevant education during training, providing care to >1 patient with gender dysphoria, and being able to provide non-judgemental care. Willingness to prescribe GAHT was higher in female-identifying participants and participants with ≥4h of education. Female- identifying clinicians were more likely to agree additional training would benefit their practice.	‘Advanced training opportunities should be available for those positioned and willing to provide such care. Future research is needed to explore trends across specialties and the effectiveness of educational efforts.’ [18]
Gahagan and Subirana-Malaret [19]	Canada	*N* = 283 LGBTQ people and *N* = 109 PCPs (of which *n* = 53 did not self-identify as LGBTQ and *n* = 56 self-identified as LGBTQ)	To explore the main health concerns and perceived pathways and barriers to primary health care for LGBTQ populations from the perspective of both a sample of LGBTQ community members and health care providers.	Anonymous online survey	LGBT population perspectives vs non-LGBTQ identifying PCP perspectives vs LGBTQ identifying PCPs (including demographics, important health topics, patient-PCP relationship/ interactions…)	Just over two thirds of LGBQ and trans respondents had experienced at least one good healthcare experience. Although there were differences in the health topics perceived the most important by LGBQ and trans groups, sexual and reproductive health was among the top three in both groups. LGBTQ identifying PCPs generally reported feeling more comfortable and knowledgeable addressing LGBTQ specific issues compared to non-LGBTQ identifying PCPs. The feeling of a lack of knowledge was more common regarding trans patients than LGBQ patients. More than half of the total PCPs had never received cultural competence training for LGBTQ populations.	‘Pathways to primary health for LGBTQ populations may be improved by addressing issues at the micro level of the individual (e.g. additional training and awareness) as well as at the macro systems level (e.g. health care systems, processes and procedures)’. [19]
Grant et al. [20]	Australia	*N* = 17 PCPs (doctors, psychologists, nurses and other allied health workers) and students (medicine, nursing and pharmacy)	To explore the knowledge and practices of clinicians and students with a view to informing health policy, training and curriculum development.	Qualitative semi-structured interviews	Understandings, approaches and awareness of TGD healthcare	Three key themes identified: (1) lack of training in TGD healthcare, (2) limited resources to support TGD patients, and (3) the importance and challenges using inclusive language.	‘The findings of this study can be used to inform design of the medical education, professional development and referral lists that should be developed to support current and future healthcare practitioners.’ [20]
Klein et al. [21]	USA	*N* = 386 military-affiliated family physicians	To assess military-affiliated family physicians’ current perception of, comfort with, and education in providing GAC, and identify differences between 2016 and 2023.	Survey	The perceived ability to provide non-judgemental care, education on GAC, willingness to prescribe GAHT, comparison of 2016 to 2023.	Military-affiliated family physicians in 2023 demonstrated more experience with and willingness to provide non-judgemental TGD-related care compared to those surveyed in 2016. In 2023, 63% of respondents would prescribe GAHT to an adult compared to 53% in 2016. Female-identifying participants and participants with ≥4 hours of education were more likely to report willingness to prescribe GAHT.	‘Advanced education should be available for those seeking training; basic education should be required for all clinicians. Research should explore trends across primary care and specialty care disciplines.’ [21]
Kremen et al. [22]	USA	*N* = 13 medical clinicians, and mental health clinicians	To explore the past and current training experiences and needs of medical and mental health clinicians providing care to TGD young adults.	Qualitative interviews	Perceived needs and barriers relating to the care of TGD young adults	Three main themes identified: (1) the need for clinicians knowledgeable about the care of TGD young adults, (2) the need and desire for reliable learning resources to train clinicians in caring for TGD young adults and their families, and (3) concerns about the impacts of the sociopolitical environment on care delivery.	‘First, additional rigorous research is needed to define best practices for the care of TGD young adults. Second, relevant clinical care competencies must be delineated for clinicians who see TGD young adults in their practice. For example, … skills for engaging in conversations about gender, creating a welcoming clinic environment, establishing a relationship of trust … Third, evidence-based and accessible training resources need to be developed to support clinicians in achieving these clinical competencies. Fourth, facilitating mentorship relationships among interested clinicians can further support professional development.’ [22]
Marconi et al. [23]	Italy	*N* = 631 GPs	To assess the knowledge and attitudes of Italian GPs towards the health of TGD individuals.	Anonymous cross-sectional online survey.	Demographics, knowledge, stigma and training experience.	General knowledge of TGD definitions and health care varied with notable knowledge gaps in cancer screening for TGD individuals. As the age of GPs decreased, the probability of providing correct responses to these questions increased. The majority (90.7%) of GPs acknowledged health care disparities experienced by TGD individuals compared to cisgender individuals. Only 5.9% had received training on TGD care, with 57.5% of these completing this training voluntarily. Nearly all (97.8%) GPs considered training on TGD health to be useful (62.6%) or fundamental (35.2%). With 40% stating they would prefer to receive this education as part of their mandatory professional development.	‘The preference expressed by these practitioners for mandatory professional development or formal educational programs highlights … a desire for a more structured and systematic approach to transgender health education, suggesting that current voluntary initiatives may not fully address the perceived educational needs.’ Efforts to improve. PCP understanding of TGD care ‘may include targeted educational initiatives, the development of evidence-based guidelines, and promoting a cultural shift within the medical community to ensure fair and respectful care for all patients.’ [23]
Potapov [24]	USA	*N* = 74 eConsults from PCPs from 11 states (56% medical or osteopathic doctors, 44% NPs)	To examine the impact of eConsults submitted to TGD specialists on the PCP experience at the point of care and on their education on TGD-related topics.	Retrospective review of de-identified registry data from the RubiconMD electronic consultation platform	Frequency of clinical question categories asked in eConsults, outcomes of eConsults reported by PCPs.	Outcomes reported by PCPs included; ‘improved care plan’ in 88% of eConsults, ‘educational’ in 50%, ‘avoided unnecessary services’ in 29% with only 2% reporting ‘no effect’ of the eConsult. GAHT questions came up in the majority of the cases (88%); followed by questions for other medical concerns such as diabetes, hyperthyroidism, cancer screening, and electrolyte abnormalities (19%)	‘eConsults may play an important role in educating PCC on TGNB care, addressing some of the knowledge gaps in TGNB care, and may also lead to improved PCC and patient experience. Additional research is needed to better understand the satisfaction of TGNB patients with eConsults and longer-term health outcomes in TGNB patients.’ [24]
Schvey et al. [25]	USA	*N* = 204 military family physicians	To assess military family physicians’ readiness to treat patients with gender dysphoria.	Online survey	Demographics, attitudes towards TGD, readiness for treating TGD patients	Of 204 participants, 95% had received fewer than three hours of TGD training with 74% having never received any at all. More than half (51%) reported they would not prescribe GAHT with 87% reporting they had not received sufficient education to provide GAHT. The majority (76%) felt they could provide nonjudgemental care to TGD patients.	‘Given that education in transgender care was associated with greater likelihood of prescribing GAHT and belief in one’s ability to provide nonjudgmental care, it will be vital to augment the training of family physicians to ensure competence and sensitivity in treating adolescents with gender dysphoria.’ [25]
Sharma et al. [26]	USA	*N* = 113 PCPs in rural Michigan	To describe existing attitudes of PCPs in rural Michigan towards LGBTQ persons, and to identify independent correlates of these attitudes.	Paper-based survey using a modified Dillman mail-out method	Demographics, PCP attitudes towards LGBTQ persons, correlates of attitudes towards gay men/ lesbian women/ bisexual men and women/ trans persons.	Forty five percent of PCPs never received LGBTQ health education and 88.5% believed that such education should be required for all PCPs. In general, PCPs had positive attitudes towards LGBTQ individuals. PCPs who had received LGBTQ health education, who believed LGBTQ education should be required and who had personal contacts that identify as LGBTQ, were associated with more favourable attitudes towards LGBTQ persons. Higher levels of religiosity, not having personal contacts who identify as LGBTQ and not having received LGBTQ health education, were associated with less favourable attitudes.	‘Educational opportunities … must be afforded to providers across the spectrum of healthcare fields to learn about the characteristics and specific needs of sexual and gender minorities. This might help improve attitudes and understanding among those who are not entirely comfortable with LGBT individuals, possibly due to their higher degrees of religious observance’. [26]
Shires et al. [27]	USA	*N* = 163 primary care clinicians in an integrated Midwest health system	To examine the extent to which primary care clinicians are willing to deliver routine care and Pap tests to TGD patients along with factors that predict willingness.	Survey conducted using REDCap	Survey included: demographics, clinical and personal exposure to TGD individuals, assessment of empathy, transphobia scale, barriers to providing care to TGD patients, willingness to provide care to TGD patients…	Of 163 participants, 85.7% and 78.6% of clinicians were willing to provide routine care to TGD patients and Pap tests to trans men, respectively. Multivariate results suggest that younger clinicians are more willing to provide routine care. Only personal experiences and biases predicted willingness to provide Pap tests to trans men. Results may overestimate willingness to care for TGD patients, due to a non-optimal response rate (53%).	‘Medical education should address professional and personal factors related to caring for the transgender population to increase access.’ [27]
Singh et al. [28]	Canada	*N* = 37 PCPs (70% physicians, 30% NPs)	To explore the impact of an electronic consultation (eConsult) service between PCPs and TGD care specialists on access to care and to explore the content of clinical questions that were asked.	Retrospective mixed methods analysis of eConsults	Qualitative analysis of questions raised by PCPs, course of action post eConsult, PCP rating the overall value of the eConsult	The median specialist response time was 1.2 days and the average time spent by specialists to respond to one eConsult was 18 min. Six major themes of PCP questions were identified: 1) interpretation/ management of abnormal bloodwork (29%), 2) change in management due to lack of desired effect/hormone levels not a target, 3) initiation of GAHT/ initial work up, 4) management of adverse effects of GAHT, 5) transition related surgery counselling and post-op complications, and 6) management of patients with comorbidities. Face-to-face referral was avoided in 32% of eConsults and 73% of PCPs received new advice for a new/additional course of action from the eConsults. Overall, 95% of PCPs rated the value of their eConsult as a 5 (excellent value) or 4.	‘Identified themes of eConsult questions provides insight into potential gaps in knowledge amongst primary care providers that could help inform future continuing education events.’ [28]
Smith and Kaplan [29]	USA	*N* = 12 family medicine residents	To assess the efficacy of a multimodal educational framework on the quality of GAC provided by residents at a large academic family medicine program.	Pilot study with a pre- and postintervention analysis	Documentation of Essential Components of GAC Pre- and Postintervention (informed consent, contraception, pronouns…) Appropriate Clinical Management in Medication Dosing and Laboratory Monitoring of GAHT Pre- and Postintervention (correct baseline and follow up labs…)	Following a multimodal educational intervention, family medicine residents demonstrated significant improvement in many aspects of GAC, including documentation and clinical management. All post-intervention charts included documentation of informed consent to initiate GAHT compared to 18% pre-intervention.	‘Further research should specifically explore faculty development in this area and expanded patient- centred quality metrics and outcomes that encompass GAC.’ [29]
Wolfe et al. [30]	USA	*N* = 15 PCPs (*n* = 11 physicians, *n* = 4 NPs)	To understand how PCPs report discussing substance use with TGD adult patients within the context of discussing GAC.	Qualitative study using semi-structured, in-depth interviews.	Demographics and approaches to substance use in the context of GAC.	Two primary themes were identified: (1) placing a focus on harm reduction, emphasising reducing the negative consequences of substance use and communicating to patients that substance use would not deter access to GAC. (2) using access to GAC as an incentive for patients to change their substance use patterns. PCPs who used approach two reported knowledge gaps/lack of training in TGD care.	‘Findings from this study carry implications for policy and clinical practice, including integrating substance use care with gender-affirming care in addition to enhancing provider knowledge around the appropriate application of gender-affirming care guidelines.’ [30]
Yip et al. [31]	USA	*N* = 220 PCPs (*n* = 137 MD/DO, *n* = 80 advanced practice providers (ANP/PA), *n* = 3 other)	To assess PCP comfort and experience with, as well as knowledge of prescribing GAHT to adult TGD patients	Anonymous online Qualtrics survey	Association between age, years in practice, or practice setting and (1) comfort in prescribing GAHT and (2) favourable statements about learning about, providing and benefitting from training in GAHT	Overall low knowledge scores regardless of age, experience, or clinical setting. Younger, early career PCPs reported being more comfortable with prescribing GAHT and had more favourable opinions regarding learning about, providing and benefitting from training in GAHT. A minority prescribe G with most reporting lack of training as a major barrier. Most PCPs agreed they would benefit from continuing education on GAHT.	‘These results underscore the need for increased educational efforts in transgender care throughout medical training for primary care providers, including CME.’ [31]
Ziegler et al. [32]	Canada	*N* = 15 PCPs (*n* = 7 NPs, *n* = 3 physicians, *n* = 3 mental health clinicians, *n* = 1 nurse, *n* = 1 pharmacist)	To understand how the implementation of primary care services for TGD individuals is undertaken and delivered by practitioners in Northern Ontario.	Convergent mixed methods design (survey and qualitative interviews)	Characteristics of the patient population, the different roles, activities and preparation of practitioners to provide this care, the process of implementation and factors which influence the program implementation	Findings indicate that there is a gap in primary care provision for TGD clients in Northern Ontario including lack of GAC as well as a lack of knowledge in PCPs.	Practice: practitioners need professional development opportunities including training about Indigenous and TwoSpirit identities. Incorporating virtual care may address some of the geographic barriers and lengthy waitlists. Research: Future research could focus on addressing the lack of knowledge among PCPs. Policy: leadership support within primary care is needed to develop TGD-specific health programs. Advocacy for primary care services for this population is vital. Policymakers need to be aware of the increased challenges and barriers to care faced by the TGD population and how these barriers are amplified in remote and rural populations.
Ziegler et al. [33]	Canada	*N* = 19 (including; physicians, NPs, nurses, allied health professionals and clinical support staff)	How is primary care for TGD individuals delivered within different primary care models in Ontario? and what activities do interdisciplinary team members engage in when delivering primary care to TGD individuals?	Exploratory multiple case study design with qualitative interviews	Participant roles and activities, barriers and enablers in the delivery of care	Participants understood their roles in providing care, however, they were not always able to describe the roles of others. Physicians and NPs were most involved in delivering primary care to TGD individuals. NPs are underutilised and not working to the full scope of practice. Team meetings and case conferences were essential in supporting interdisciplinary collaboration. The main barrier to team collaboration was difficulty accessing services from other team members. Practitioners struggled to identify other health services which were safe spaces for TGD individuals. A lack of education was identified and PCPs were unaware of where to obtain training. Practitioners identified guidelines, training and conferences as the sources to develop competence.	‘Results support the development of educational initiatives that include the delivery of care to transgender individuals. More research is needed to focus on collaboration in teams providing care for transgender individuals and impact on health outcomes and patient experiences’. [33]

*Note:* LGBTQ+: lesbian, gay, bisexual, transgender, or queer; SOGI: sexual orientation and gender identity; GP: general practitioner; TGD: transgender and gender diverse; PCP: primary care provider; NP: nurse practitioner; GAHT: gender-affirming hormone therapy; GAC: gender-affirming care; Trans: transgender.

### Step 5: Collating, summarising and reporting results

Data were collated, presented and reported in the results section (see Table 3). Major themes were developed using Braun and Clarke’s framework for thematic analysis [11] using NVivo software. Initial coding and theme generation were conducted by KLK and further refined through collaborative reflection with the wider research team (DC, MCVH, GMC) to reach the final thematic structure.

## Results

**Figure 1.**
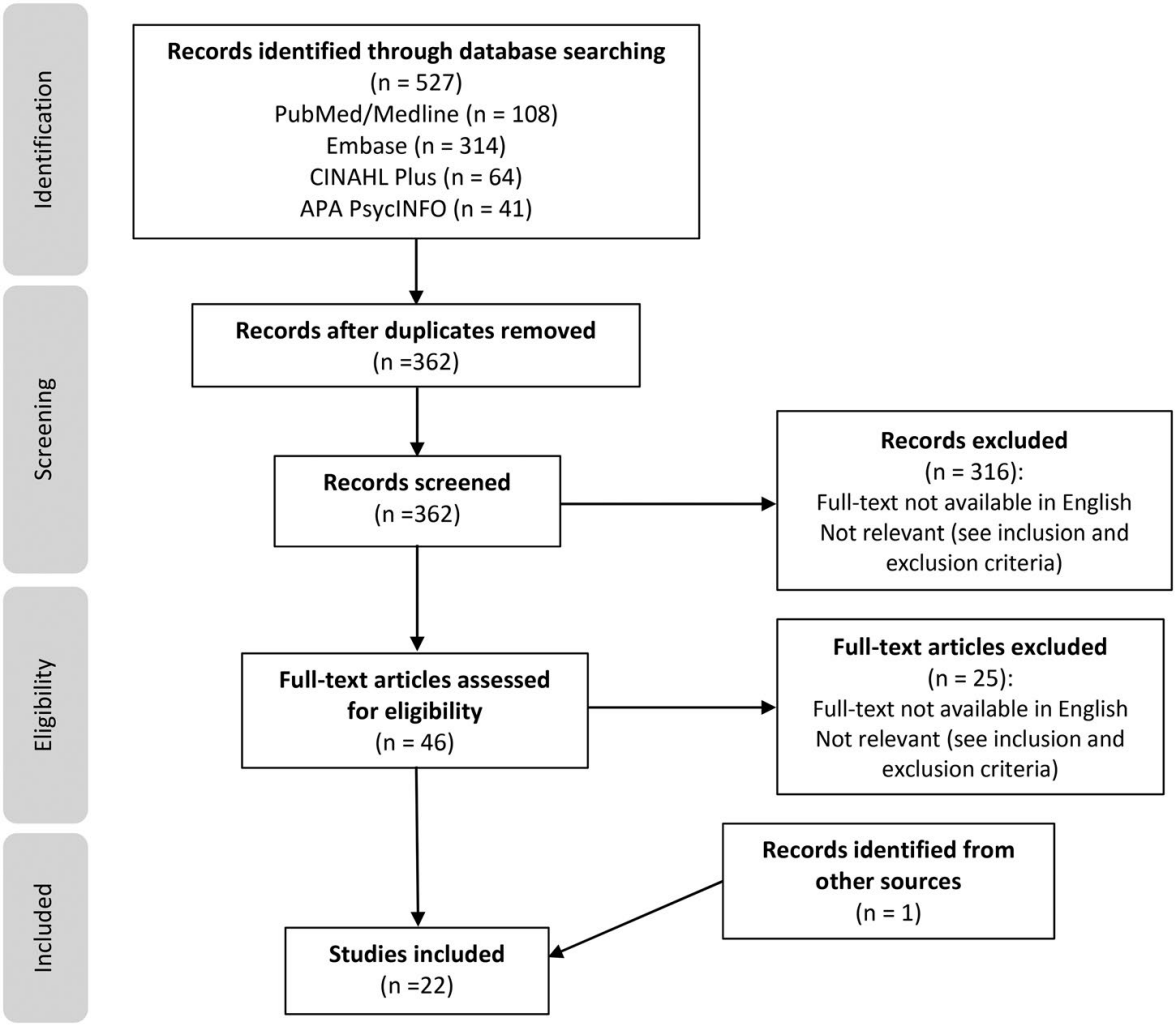
PRISMA-ScR flow diagram.

Initial database searching identified 527 studies. Following screening and the addition of one paper identified by an external reviewer [20], 22 papers were included in this scoping review. The search, identification and selection process are summarised in the ‘Preferred Reporting Items for Systematic Reviews and Meta-Analyses Extension for Scoping Reviews’ (PRISMA-ScR) [34] diagram (Figure 1).

### Study characteristics

As detailed in Table 4, the majority of studies were conducted in North America (USA [12,15,17,18,21,22,24–27,29–31] and Canada [13,16,19,28,32,33]), primarily utilising survey [12,15,16,18,19,21,23,25–27,31] or qualitative [13,14,20,22,30,33] methodologies. Sample sizes varied significantly, from 10 (14) to 1245 (12) participants. While all studies included PCPs, specific cohorts varied, ranging from physician-only samples [13,18,21,23,25,27,29] to multidisciplinary teams.

**Table 4.** Characteristics of included studies (*n* = 22).

Characteristic	Categories	*n* (%) or range	References
Study design	Survey	11 (50.0%)	[12,15,16,18,19,21,23,25–27,31]
	Qualitative (interviews)	6 (27.3%)	[13,14,20,22,30,33]
	Mixed methods	3 (13.6%)	[17,28,32]
	Retrospective review	1 (4.5%)	[24]
	Pilot study with pre- and post-intervention analysis	1 (4.5%)	[29]
Country	USA	13 (59.1%)	[12,15,17,18,21,22,24–27,29–31]
	Canada	6 (27.3%)	[13,16,19,28,32,33]
	Italy	1 (4.5%)	[23]
	Netherlands	1 (4.5%)	[14]
	Australia	1 (4.5%)	[20]
Study population	Physicians only	7 (31.8%)	[13,18,21,23,25,27,29]
	PCPs (physicians, NPs, allied health professionals, clinical support staff, etc.)	6 (27.3%)	[15,22,26,31–33]
	Physicians and NPs	4 (18.2%)	[16,24,28,30]
	TGD individuals and PCPs	2 (9.1%)	[14,17]
	LGBTQ-identifying individuals and PCPs	1 (4.5%)	[19]
	PCPs and students	1 (4.5%)	[20]
	Primary care practices	1 (4.5%)	[12]
Sample characteristics	PCP sample size across studies	10–1245	–
	Studies reporting PCP gender identity	11 (50.0%)	[13,15,16,18–21,25,26,30,31]
Sample limitations	Low survey response rate	6 (27.3%)	[12,15–17,26,27]
	Self-selected sample	3 (13.6%)	[13,20,29]

*Note:* PCP: primary care provider; NP: nurse practitioner; TGD: transgender and gender diverse; LGBTQ+: lesbian, gay, bisexual, transgender, or queer.

### Integrated findings

Thematic analysis of the included articles resulted in the identification of two themes, which are outlined below.

### Theme 1: PCP knowledge of TGD adult healthcare profiles & needs and educational deficits

#### Lack of TGD-specific education

Seventeen papers [12–20,22,23,25–27,31–33] highlighted the amount of TGD-specific training received by PCP participants. Each of these reported low levels of TGD-specific training received during their degree and in postgraduate training. Marconi et al. [23] reported that only 5.9% of Italian general practitioners (GPs) had received training on TGD issues. Carroll et al. [15] found that TGD-specific training ranged from 0 to 10 or more hours, with 29% of PCPs having received six or more hours. Five papers [13,14,19,22,32] highlighted that the majority of PCPs who had received education completed it on their own initiative. The main barriers to sourcing TGD-specific education were lack of faculty with expertise in the area, poor clinical exposure in training [15], unaware of where to obtain training [20,33], cost and/or lack of funding [22,32], location [32], lack of professional continuing education credits for TGD training completion and lack of easily accessible training/mentorship [22].

#### PCP knowledge gaps identified

Ten of the included papers [14–16,22–24,27–29,31] identified knowledge gaps among PCP participants. The most prevalent being gender-affirming hormone therapy (GAHT), which included initiation, consent, chart documentation, adverse effects, management of hormone targets, management of abnormal bloodwork, correct labs, etc. Questions regarding breast/prostate/cervical cancer screening recommendations were poorly answered, with Carroll et al. [15] finding that only 35% were aware that specific TGD breast cancer screening recommendations existed. Knowledge gaps extended to reproductive health, with one study finding that less than one-third of PCPs correctly identified that feminising hormones alone are not a reliable form of contraception [15].

#### Positive impact of TGD-specific education

Five papers [15,18,21,23,25] compared PCPs who had received TGD-specific education against those who had not. These papers highlighted that PCPs with previous TGD-specific training were more likely to answer TGD-health-related questions correctly, to regard training as important, to prescribe GAHT and were more aware of TGD cancer screening guidelines.

Three papers [24,28,29] evaluated specific educational interventions, two of which explored eConsults [24,28]. Overall, eConsults were rated highly among the PCPs, with most scoring four/five out of five in both studies. These consultations provided clinical advice and reduced the need for specialist referrals. While most PCPs found them educational, a minority reported that the eConsults had a negligible impact. The third paper [29] utilised chart reviews to measure the efficacy of a lecture and case-based discussion session on TGD healthcare. Post-intervention chart reviews improved across nearly all domains.

#### TGD-specific education going forward

Different sources of TGD-specific education were identified in six papers [14–16,22,32,33]. These included lectures, guidelines, in-clinic training, conferences, expert groups, mentorship from peers, etc. PCPs in nine papers [15,16,18,22,23,26,28,31,33] highlighted the importance of TGD-specific education and expressed interest in further training, with some believing that it should be required for all PCPs [26]. One study reported that 67% of PCPs desired TGD-specific medical education [16]. When asked how they would like to receive TGD education going forward, Marconi et al. [23] found that GPs would prefer to receive TGD-specific education as part of mandatory professional development (40%) and/or mandatory formal educational program (32.7%). PCPs identified lectures, in-person conferences, online resources, continuing medical education (CME) courses and webinars as the most effective formats for future training [15,16].

### Theme 2: PCP attitudes and opinions

#### Comfort caring for TGD individuals

Eight papers [14,16–18,20,21,25,27,31] examined the comfort of PCPs when caring for TGD individuals. Generally, most PCPs were comfortable providing routine care to TGD individuals but reported a lack of comfort providing TGD-related care. Christopherson et al. [16] found that while 95.8% of PCPs felt comfortable providing non-TGD-related care to TGD patients, only 30.3% felt comfortable providing TGD-related care. With regard to prescribing GAHT, Klein et al. [21] noted that 63% would prescribe it either independently, with further education or with a more experienced clinician. Conversely, commonly reported barriers to providing GAHT included ethical concerns, lack of comfort, lack of training, outside scope of practice, concerns about liability, lack of TGD patients in the practice and patient adherence issues [18,21,31].

The political climate also dictates PCP comfort when providing care to TGD patients. One study explored how the political environment in specific US states can act as a barrier due to changing legislation, influx of TGD patients from less supportive states and the fear of threats [22]. On a practice level, location appears to impact GAC with high performing practices in states with high LGBTQ+ Equality Scores and lower scores in rural practices [12].

As seen in Table 5, comfort levels varied significantly across PCP subgroups, with subsequent differences in clinical impact. The frequency of TGD patient exposure varied from study to study, with PCPs who had cared for at least one ranging from 33.1% to 100% [15–18,22,23,25,27,31,32]. Two papers explored the presence of transphobia among the PCP sample and reported low on average [17,27].

**Table 5.** Comfort levels between different subgroups of PCP.

PCP subgroup	Clinical impact	References
Increased exposure to TGD individuals	More favourable attitudes towards TGD individuals More likely to be willing to prescribe GAHT Increased awareness of TGD-specific cancer screening guidelines More likely to be willing to provide Pap smears to trans men More confidence when providing care to TGD individuals	[15,18,20,27]
LGBTQ-identifying	More likely to be willing to prescribe GAHT More awareness of TGD-specific cancer screening guidelines (72% versus 33% that did not identify as LGBTQ+) Greater understanding of TGD healthcare	[15,18,20]
Female-identifying	More likely to be willing to prescribe GAHT More likely to regard TGD-specific training as beneficial Lower odds of reporting ethical concerns as a barrier to prescribing GAHT	[18,21]
Younger age	More likely to be willing to provide routine care to TGD individuals [27] More likely to feel comfortable to prescribe and learn about GAHT More likely to correctly answer questions regarding TGD health More likely regard TGD-specific training as important	[15,23,27,31]
Early career (≤10 years)	More likely to report being comfortable prescribing GAHT	[31]
Non-academic setting	More likely to report being comfortable prescribing GAHT	[31]
High religiosity	Less favourable attitudes towards TGD individuals	[26]
Transphobia	Less willing to provide pap smears to trans men	[27]
Ethical/religious/moral reservations	Less willing to provide any care to TGD individuals	[16]

*Note:* PCP: primary care provider; TGD: transgender and gender diverse; GAHT: gender-affirming hormone therapy; Trans: transgender; LGBTQ+: lesbian, gay, bisexual, transgender, or queer.

#### PCP perceptions

Ten papers [13,14,19–23,25,30,32,33] explored how PCPs perceive TGD healthcare compared to cisgender healthcare. A debate emerged regarding the importance of knowing patients’ Sexual orientation and gender identity (SOGI) in one study [13]. Some PCPs argued that it did not matter as they treat all patients the same/equally with others argued that it is necessary for holistic care as it may impact the patients’ lives and health. There was also a range of responses when PCPs were asked about the differences between the health concerns of LGBTQ-identifying and non-identifying populations. While some saw no difference between primary care for LGBTQ and primary care for any other patients, others categorised them as ‘similar’ or ‘completely different’ [13,19]. LGBTQ-identifying PCPs were more likely to agree that LGBTQ+ populations have distinct health concerns differing from heterosexual/cisgender populations [19]. Marconi et al. [23] found that 90.7% of Italian GPs surveyed acknowledged that disparities exist in the healthcare received by TGD individuals compared to cisgender individuals.

Perceived barriers to care from a PCP perspective included struggling to find knowledgeable and accepting external providers for referrals, coordinating multidisciplinary care, reluctance in prescribing GAHT due to state laws and a general lack of societal awareness [13,14,20–22,30,32,33]. Perceived facilitators to care included team-based discussions and collaboration, patient documentation which includes gender identity, development of an ‘expert’ referral list and hearing about TGD lived experiences of healthcare (Table 6) [14,20,22,25,32,33].

**Table 6.** Summary of barriers and facilitators to care of TGD individuals from the PCP perspective.

	Barriers to care	Facilitators to care	References
Individual (PCP attributes)	Transphobia Higher levels of religiosity Ethical/religious/moral reservations	Increased personal exposure to TGD individuals Younger age LGBTQ-identifying Female-identifying Early career (≤10 years)	[15,16,18,20,21,23,26,27,31]
Interpersonal (clinical skills)	Low comfort providing TGD-related care Clinical knowledge gaps - GAHT, TGD-specific cancer screening, reproductive health Unawareness of TGD-specific guidelines ‘Identity-blind’ philosophy Fear of liability and/or threats	High comfort providing routine care Learning from TGD lived experiences	[14–18,20–25,27–29,31]
Institutional (clinic & education)	Lack of TGD-specific training/education Unclear scope of practice regarding GAHT Poor referral networks Underutilisation of NPs	Receiving TGD-specific training/education eConsults and specialist mentorship Multidisciplinary teams Gender-inclusive patient documentation Development of ‘expert’ referral lists for different areas	[12–33]
Systemic (societal & political)	Unsupportive political climates Rural location Restrictive state laws General societal transphobia	Regions with high LGBTQ+ equality score	[12,14,22,32]

*Note:* PCP: primary care provider; TGD: transgender and gender diverse; LGBTQ+: lesbian, gay, bisexual, transgender, or queer; GAHT: gender-affirming hormone therapy; NP: nurse practitioner.

#### Role of PCPs in TGD care

Four papers [17,30,32,33] discussed the role of PCPs when working with TGD individuals. Participants in one study believed that ‘providing care to [the TGD] population was primary care and within the scope of primary care practitioners’ [32]. DeBrosse et al. [17] found that 59% of PCPs felt that prescribing GAHT was their professional responsibility, with 76% agreeing that endocrinology referral is not always necessary for GAHT. One study [33] found that when working in a multidisciplinary team, PCPs could describe their own role with TGD patients but could not describe other team members’ roles. It also found that nurse practitioners (NPs) were ‘underutilised and not working to the full scope of practice’ [33]. Wolfe et al. [30] explored the role of PCPs in discussing substance use with regard to GAHT. It found that PCPs typically had two different strategies: using GAHT as an incentive to change substance use or communicating harm reduction to reduce negative consequences of substance use while not hindering access to GAHT.

## Discussion

### Main findings

This scoping review identified two primary themes: (1) PCP knowledge of TGD adult healthcare profiles and needs and educational deficits and (2) PCP attitudes and opinions. These themes directly address both research questions:

#### PCP perspectives of experiences with TGD adults

Internationally, PCP experiences are characterised by a significant lack of TGD-specific education [12–20,22,23,25–27,31–33], with major knowledge gaps identified in GAHT and TGD cancer screening recommendations [14–16,22–24,27–29,31]. A desire for future TGD-specific education for PCPs was evident [15,16,18,22,23,26,28,31,33]. Most PCPs reported a lack of comfort providing TGD-related care despite being comfortable providing routine care to TGD patients [14,16–18,20,21,25,27,31]. Furthermore, the PCPs’ role remains unclear regarding prescribing GAHT, within multidisciplinary teams and managing substance use alongside GAC [17,30,32,33].

#### Main barriers and facilitators to care

Barriers and facilitators to care may be split up into four groups: (1) Individual (PCP attributes), (2) Interpersonal (clinical skills), (3) Institutional (clinic & education) and (4) Systemic (societal & political). The lack of TGD-specific education [12–19,22,23,25–27,31–33] constitutes a primary barrier, negatively impacting general comfort caring for TGD individuals, awareness of TGD cancer screening guidelines and willingness to prescribe GAHT. Conversely, facilitators include receiving TGD-specific education [15,18,21,23,25], increased exposure to TGD individuals [15,18,20,27] and eConsults [24,28].

### Strengths and limitations

To our knowledge, this is the first literature review focused on TGD primary care from the perspective of the PCP. A scoping review methodology was therefore appropriate, as it is well-suited to mapping and describing literature in emerging research topics. The application of Arksey and O’Malley’s five-stage framework [10], PRISMA-ScR framework [10] and Braun and Clarke’s thematic analysis guidelines [11] ensured a transparent and rigorous review process.

However, this study has several limitations. Following scoping review protocols, we did not critically appraise the evidence, as our goal was to map the breadth of literature rather than filter by scientific rigour. Additionally, despite our comprehensive approach, some relevant literature may have been missed due to specific search parameters or eligibility criteria employed. Although this review did not restrict by geographical location, the final dataset represented only five countries; one study originated from Australia [20] and two from Europe [14,23], while the remainder originated from North America [12,13,15–19,21,22,24–33]. This concentration suggests a significant geographic bias in current literature. Furthermore, many selected studies faced limitations such as low sample sizes, low response rates and self-selected samples [13,15–18,20,22,26,27,29,31,32]. These factors introduce potential non-response or sampling biases, which may limit the generalisability of the findings to the broader PCP population. Despite this, the 22 included studies provide a comprehensive overview of current literature.

### Implications for policy, practice and research

This study’s findings include many implementable facilitators for the care of TGD individuals for PCPs. On a policy level, regulatory bodies should integrate mandatory TGD-specific healthcare modules into undergraduate and postgraduate medical curricula to address the significant educational deficits identified. Standardised GAC guidelines for primary care may help existing PCPs, especially with GAHT, referrals and cancer screening. Furthermore, addressing systemic barriers requires policy protections for PCPs against liability and restrictive legislative climates when providing GAC.

eConsults should be implemented and utilised on a practice level as they are effective in providing specialist guidance and reducing the amount of unnecessary referrals [24,28]. Strengthening multidisciplinary teams and peer collaboration will facilitate more holistic care for TGD individuals. Practice health records should include SOGI data to facilitate affirming environments within primary care.

Finally, future research is needed to identify the most effective formats for TGD-specific training and to evaluate whether these interventions improve care from the patient perspective. Targeted education increases PCP comfort, reduces specialist waitlists and improves healthcare uptake among TGD populations [15,18,21,23,25]. Many TGD policies and screening guidelines are available, but large numbers of PCPs are unaware of them or unaware of where to find them [15]. By receiving TGD-specific education, these guidelines and policies are more likely to be utilised. Future research should also investigate why younger and female-identifying PCPs demonstrate greater proactivity in GAC to help mirror these positive clinical attitudes across the broader medical workforce.

## Conclusion

This scoping review provides a comprehensive map and description of the existing literature regarding PCP perspectives on experiences with TGD adults. The most apparent barrier to care identified is a pervasive lack of TGD-specific healthcare knowledge, stemming from educational deficits. Included studies demonstrate that education programmes and digital interventions, such as eConsults, significantly improve PCP comfort and the quality of patient care.

Moving forward, the focus must shift from identifying clinical deficits to implementing multi-level facilitators (individual, interpersonal, institutional and systemic) to bridge the gap between policy and practice. Future research should prioritise identifying effective formats for TGD-specific training, investigating demographic drivers that foster proactive GAC and ensuring a broader geographical representation of data. Ultimately, integrating TGD-specific education into medical training is essential to ensuring equitable and accessible primary care for TGD individuals globally.
